# The Old Port of Dubrovnik

**DOI:** 10.3201/eid0801.030100

**Published:** 2002-01

**Figure:**
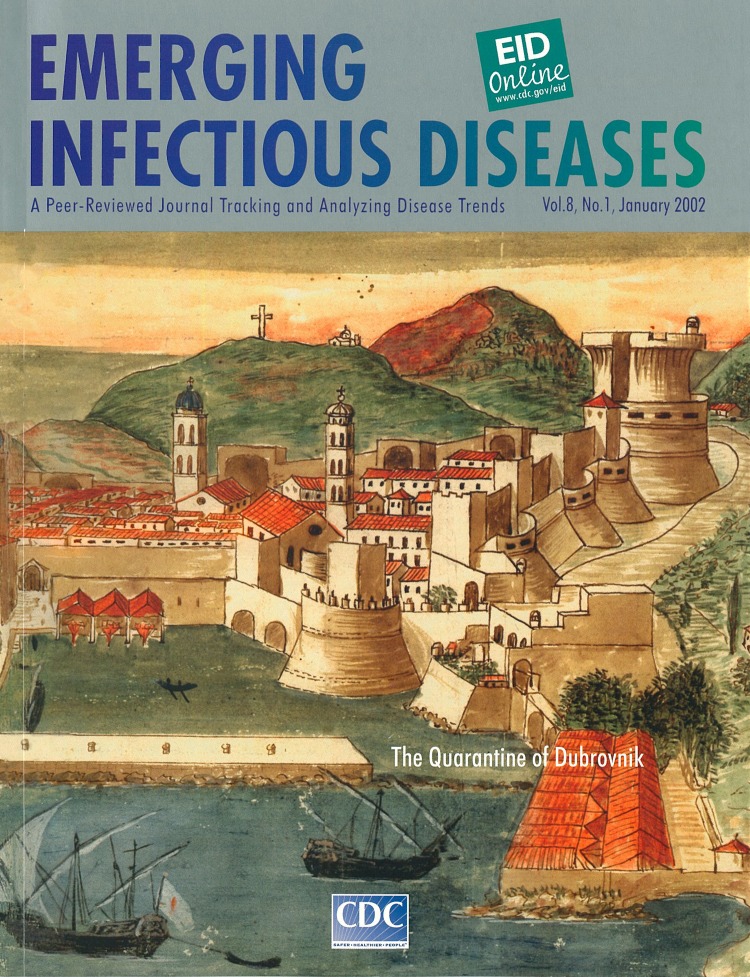
**The Old Port of Dubrovnik (watercolor, 18th century).** Anonymous.Provided courtesy of Dr. Andreja Tambic-Andrana (CK)

This 18th-century painting depicts quarantine activities at the port of Dubrovnik, for many centuries one of the largest cultural centers in Croatia and a major trading center on the Dalmatian coast. Because trade with the East and the West was the driving force behind the development of Dubrovnik, suspension of trade during plague epidemics in the 14th century would have been disastrous for the city. Therefore, on July 27, 1377, the Great Council of Dubrovnik introduced in a decree a measure that would both protect against plague epidemics and free trade with eastern countries from which these epidemics usually spread. The text of this decree can be seen in Volume 78, chapter 49 of the Liber Viridis. The original document, which is kept in the Archives of Dubrovnik, states that before entering the city, newcomers had to spend 30 days in a restricted location awaiting to see whether the symptoms of plague would develop. Later on, isolation was prolonged to 40 days and was called quarantine.

The word quarantine, used to describe isolation to prevent spread of infection, comes from the Latin word "quaranta," meaning 40, because the isolation lasted for 40 days. Along with Venice and Milan, Dubrovnik was among the first cities in the world to introduce isolation as a measure to control the spread of infectious disease and the first city to have a documented organization of quarantine. Over the centuries, other epidemic diseases (leprosy, smallpox, dysentery) were recorded in the archives of Dubrovnik, and other specialized institutions (e.g., leprosaria) were organized outside the city. In 1590, quarantine activities were moved to the complex of houses near the east city gates, as can be seen on the painting (red-roofed houses at the bottom right of the painting). Isolation proved to be effective; none of the plague epidemics that occurred in centuries to come was as devastating as Black Death, which spread throughout the world in the mid-14th century. Isolation is probably the greatest achievement of medieval medicine, and the quarantine of Dubrovnik is an important development in the medical heritage of Dubrovnik and Croatia. 

